# Incidence of tinnitus in mp3 player users

**DOI:** 10.1590/S1808-86942011000300004

**Published:** 2015-10-19

**Authors:** Ricardo Rodrigues Figueiredo, Andreia Aparecida de Azevedo, Patrícia Mello de Oliveira, Sandro Pereira Vasconcellos Amorim, Artur Guedes Rios, Vanderlei Baptista

**Affiliations:** 1 Master's degree in general surgery, area of concentration - ENT, Rio de Janeiro Federal University (Universidade Federal do Rio de Janeiro). Adjunct professor and head of the ENT unit, Valença Medical School (Faculdade de Medicina de Valença), RJ; 2 Physician, otorhinolaryngologist at OTOSUL (Otorrinolaringologia Sul-Fluminense); 3 Audiologist, speech therapist at OTOSUL (Otorrinolaringologia Sul-Fluminense); 4 6^th^-year medical undergraduate, Valença Medical School, RJ. Scholarship from the scientific initiation program, Valença Medical School; 5 6^th^-year medical undergraduate, Valença Medical School, RJ. Scholarship from the scientific initiation program, Valença Medical School; 6 6^th^-year medical undergraduate, Valença Medical School, RJ. Scholarship from the scientific initiation program, Valença Medical School

**Keywords:** audiometry, hearing loss noise-induced, music, otoacoustic emissions spontaneous, tinnitus

## Abstract

Exposure to loud noise is one of the main causes of tinnitus.

**Aim:**

To analyze the incidence of tinnitus in mp3 player users and non-users.

**Material and method:**

One hundred subjects aged from 15 to 30 years were enrolled, 54 of them were regular mp3 player users and 46 were not. Patients with continuous tinnitus for at least 6 months completed the Tinnitus Handicap Inventory (THI) and were tested with high frequency audiometry and transient-evoked otoacoustic emissions (TAOE).

**Study design:**

A cross-sectional cohort study.

**Results:**

The incidence of tinnitus in non-users was about 8%; in mp3 player users it was about 28 %, a statistically significant difference. Hearing thresholds at 8kHz were significantly higher in tinnitus patients that used mp3 portable players. TAOE were reduced at 2 kHz in the users group. No statistically significant difference was found in the THI scores between the two groups.

**Conclusion:**

Tinnitus was more frequent in teenagers and young adults who regularly listen to mp3 music in players. Moreover, the incidence of tinnitus among mp3 player users was associated with higher hearing thresholds at 8 kHz and lower TOAE at 2 kHz. Clinicaltrials.gov registration number: NCT 01187251

## INTRODUCTION

Several authors consider noise induced hearing loss (NIHL) due to high sound pressure levels as one of the main causes of tinnitus.^1^ Continuous exposure to noise - in work settings or not - may result in injury to hair cells and the auditory nerve by several mechanisms,^2^ such as glutamate excitotoxicity.^3^ Although a few recent studies have focused on recreational exposure to music - mainly in teenagers - most papers and government guidelines have emphasized exposure to industrial noise in a work setting.^4^

The mp3 system is the most frequently used codec to compress audio files; it uses variable compression rates (bitrates) to reproduce the original file quite faithfully.^5^ The mp3 compression algorithm consists of three steps:

1 Fragmentation of the original file into uniformly sized subunits (frames).
2 Quantification of spectrum components and efficient allocation.
3 Frame compression using Huffman's coding system.^6^

Information in the original file is lost in step 2, reducing the quality of sound in mp3 files compared to the original file. Mathematical models in the coding system establish the most significant frequencies (most perceived by the auditory system) and reserve fewer bytes for other frequencies. The overall effect is more sound compression at middle frequencies.^5^

Personal stereo devices [mp3 players] have become increasingly popular in the last decade. The ease of downloading music from the internet has made large amount of musical information available a minimal or zero cost; the mp3 format is ideal in this context.^7^ The quality of devices and headphones has also increased, which makes it possible to increase the volume and enjoy correction of distortion; thus, users may listen to loud music without bothering neighboring persons. Many users listen to music in noisy environments, such as inside public transport vehicles, and end raising the volume still further. Earplug type phones are preferred,^7^ as they generate more sound pressure on the tympanic membrane. Since these types of earphones do not seal the outer ear canal perfectly, users find it necessary to raise the volume.

At the same time, the scientific community and society in general have grown concerned with the possible harmful effects of frequent use of personal stereo devices. A study sponsored by the American Speech Language Hearing Association (ASHA) revealed that 61% of teenagers own such devices,^8^ and 51% of students in North-American secondary schools had hearing loss symptoms. An interesting finding in this study was that most students prefer high volumes, whereas adults tend to use moderate volumes. Another study undertaken in the Netherlands showed similar results, especially in male teenagers.^9^

The most commonly used diagnostic method for the diagnosis and follow-up of NIHL is pure tone audiometry; otoacoustic emissions (OAE) and high frequency audiometry may be useful for an early diagnosis.^10^

Tinnitus is the patient's perception of sounds or noises in the absence of corresponding external sound. Estimates in the US indicate that 35 to 50 million people have tinnitus; the impact is significant in 5 to 20% of cases.^11^ Although its pathophysiology has not been clarified, most researchers think that tinnitus arises as a result of neuroplastic changes in peripheral - and mainly central - auditory pathways as a result of peripheral injury (deafferentation).^12^ Causes of hearing loss, such as NIHL, metabolic disease, genetic conditions, and vasculopathies have been described as the most important etiologies of NIHL;^1^ tinnitus has also been described as one of the main and earliest symptoms of NIHL.^2^

As far as we have been able to review, the role of constant use of personal stereo devices in the onset of tinnitus has not been studied. The main purpose of this study was to establish whether the incidence of tinnitus in teenagers and young adults differed between users and non-users of personal stereo devices, and to verify if there were changes in tone thresholds - including those at high frequencies - and in otoemissions.

## PATIENTS AND METHODS

The sample consisted of 100 subjects aged from 15 to 30 years. These were students, teachers, and staff of secondary schools who were invited to participate in this study, which took place from June 2009 to March 2010. Exclusion criteria were tinnitus of muscular or vascular causes, temporomandibular joint conditions, outer and middle ear diseases (not including wax, which was removed, if present), professions and other activities in which there was regular exposure to noise, and intermittent tinnitus within the past 6 months. Group 1 consisted of regular users of personal stereo devices (regular use being defined as 1 hour daily during at least 1 year), and group 2 consisted of non-users.

After the subjects or caretakers signed a free informed consent form, the procedure consisted of a specific medical history with information about profession, number of hours spent daily listening to music using mp3 players, hypoacusis, fullness of the ear, dizziness, tinnitus, concomitant diseases and medication use, family history of hearing loss, smoking, and consumption of caffeine and alcohol. Information on tinnitus included its type, side, periodicity, and duration.

The following tests were done (in acoustic booths) after 24 hours of withdrawal of music and loud noise.

- high frequency audiometry (Amplaid A177 Plus, Italy)
- immittance testing (Amplaid 750, Italy)
- transient otoacoustic emissions (TOAE) (Oto-Read, Interacoustics, Denmark)

TOAE were evoked with non-linear clicks at 74 dB SPL in half-octave bands; the central frequencies were 0.7, 1, 1.4, 2, 2.8, and 4 kHz. The choice of using TOAE was because they represent bands consisting of several frequencies, with more intensity at a given frequency, thereby reproducing compound musical tones more closely.

Patients with tinnitus also filled in the Tinnitus Handicap Inventory (THI) - its Brazilian Portuguese validated version.^13^

The Mann-Whitney test was applied for comparing quantitative variables (numerical) between both groups. The chi-square (c^2^) test was used for comparing qualitative variables (categorical). Non-parametric methods were applied because the variables were not distributed normally, considering data dispersion and an asymmetric distribution. The significance level was 5%. A commercial software for statistical studies was used (SAS® System, version 6.11, SAS Institute, Inc., Cary, NC). The institutional review board for research on human beings approved the study.

## RESULTS

**Figure 1:**
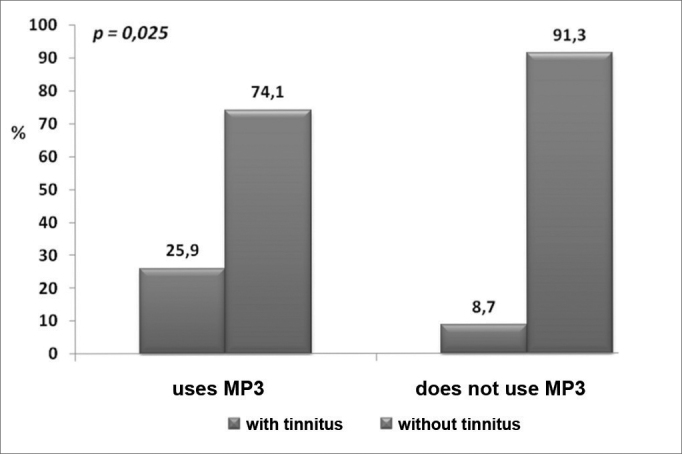
Incidence of tinnitus in both groups.

Table 1 shows the characteristics of the sample in both groups. The chart on Figure 1 presents the incidence of tinnitus. A feature was the higher incidence of inner ear symptoms - including tinnitus - in Group 1. The THI scores were similar in both groups (*p*=0.99). Table 2 shows that the incidence of tinnitus was not related with duration of use (daily hours and years).

**Table 1:** Analysis of clinical variables according to use of personal stereo devices.

Variable	Group 1 (uses personal stereo devices) (n = 54)		Group 2 (non-users of personal stereo devices) (n = 46)		*p* value
	n	%	n	%	
Male gender	22	40.7	24	52,2	0.25
Hypoacusis	18	33.3	3	6,5	0.001
Tinnitus	14	25.9	4	8,7	0.025
Fullness	13	24.1	4	8,7	0.041
Dizziness	16	29.6	6	13,0	0.046
Other diseases	6	11.1	5	10,9	0.96
Family history of deafness	12	22.2	7	15,2	0.37
Smoking	2	3.7	2	4,4	fc
Excessive use of caffeine	23	42.6	13	28,3	0.13
Alcohol	8	14.8	10	21,74	0.36
Age (years)	17.5 ± 2.2		21,3 ± 4,7		< 0.001
THI (score)	15.4 ± 11.9 (12)		16,0 ± 13,8 (12)		0.99

Age expressed as mean ± standard deviation. Compared using Student's t test.

THI expressed as mean ± standard deviation (median). C Compared using the Mann-Whitney test; 14 patients in group 1, and 4 patients in group 2. fc: few cases < 5 smokers.

**Table 2:** Analysis of variables in patterns of use of personal stereo devices relative to tinnitus.

Variable	tinnitus	n	Mean	SD	Median	Minimum	Maximum	*p* value
Daily hours using	yes	14	2.50	1.34	2	1	6	0.48
	no	40	3.05	2.58	2	1	14	0.48
Time using (years)	yes	14	3.00	1.80	2.5	1	7	0.51
	no	40	2.63	1.51	2	1	5	

SD: standard deviation

**Figure 2:**
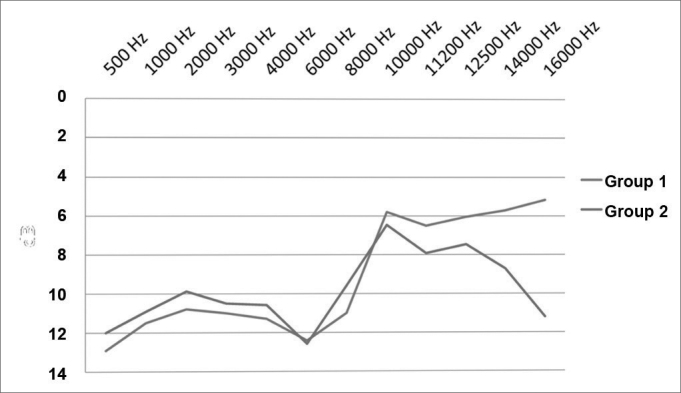
Mean value of auditory thresholds (dB HL × Hz) in both groups.

The chart in Figure 2 shows tone thresholds up to 16 kHz in both groups; higher thresholds were seen at 14 and 16 kHz in group 2. A separate analysis of patients with or without tinnitus in group 1 revealed that 8 kHz thresholds were higher in ears with tinnitus (*p*=0,025).

**Figure 3:**
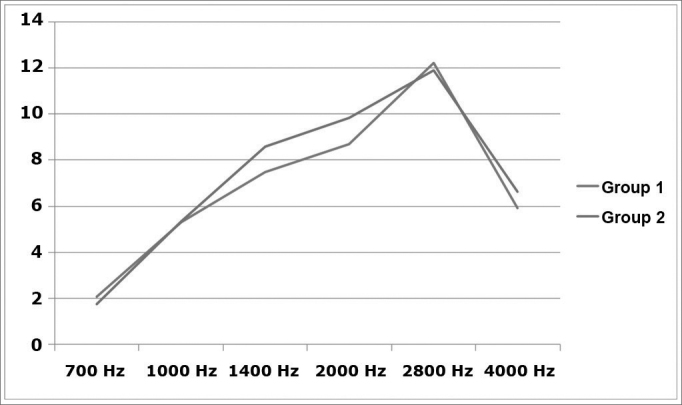
TOAE (dB SPL × Hz) in both groups.

The chart in Figure 3 shows TOAE in both groups. TOAE were decreased at 2 kHz in group 1, compared to group 2.

## DISCUSSION

There has been a growing concern in recent years throughout society about the effects of loud music and noise.^14^ Although the main purpose of this study was not to investigate this point, age differences between the two groups (*p*<0.001) underlines this fact, suggesting that adults are more concerned about the use of personal stereo devices than teenagers; studies have shown that teenagers appear unconcerned with the volume of sound.^7^, ^9^ The present study was unable to establish a direct relationship between the number of hours per day/duration of use in years and the incidence of tinnitus; the volume itself was not studied. Furthermore, the severity of NIHL varies among individuals exposed to similar levels of noise.^2^

Our data concur with other studies in that hearing loss in teenagers and young adults that use personal stereo devices is not clinically significant; frequency thresholds that are commonly affected in NIHL (3, 4, and 6 kHz) remained unaltered.^14^ Although a few studies have described the effects of loud music, the sample in most of these studies consisted of professional musicians or disk-jokeys.^15^ A South Korean study reported higher thresholds at 4 kHz in teenagers that had used personal stereo devices for more than 5 years and/or over 15 hours per day;^16^ these numbers are significantly higher than those in our study. However, even in that study, thresholds were within normal limits (below 25 dB HL). Higher thresholds at 14 and 16 kHz in group 2 were probably due to its higher age range; as previously described, high frequency hearing tends to deteriorate after adolescence.^17^

According to our results, a decrease in TOAE at 2 kHz in users of personal stereo devices may reflect initial stages of cochlear injury that precede changes in tone thresholds. A few studies have described an initial decrease of OAE as subclinical cochlear disease,^18^ which might precede tone threshold changes by a few years if exposure to noise persists. Bhagat & Davis (2008) measured distortion product OAE in normal-hearing adults and found significantly lower levels following exposure to music from personal stereo devices.^19^ Based on these facts, and given that NIHL is irreversible but preventable, otoemissions may be useful in the early detection of noise-associated cochlear injury, as long as alerts are given to children and parents, as has been recommended.^8^ On the other hand, the most affected frequencies in NIHL according to the literature are 4.6 and 8 kHz,^2^ which differ from the affected frequency we found (around 2 kHz). It is possible that this frequency matches the frequency bands of highest sound pressure in mp3 files.^5^ Additionally, other studies have found decreased otoemission amplitudes at 2 kHz in tinnitus patients exposed to noise, which has not yet been explained; this suggests that other still unknown factors may be present in the genesis of tinnitus in patients exposed to noise.^20^ In the present study, TOAE were chosen because they represent ear responses to frequency bands that are more similar to music sounds rather than pure tones or distortion products. Other studies have satisfactorily used TOAE to assess hearing in patients exposed to noise.^21^

The incidence of tinnitus in users of personal stereo devices has not yet been established, as far as we have been able to review. Our data show a clear relationship between the presence of tinnitus and regular use of personal stereo devices (*p*=0.025); the same applies to hypoacusis (*p*=0.001), dizziness (*p*=0.046), and fullness of the ear (*p*=0.041). These are inner ear symptoms in a young population without other predisposing factors (diseases, family history of deafness, other types of noise exposure, alcoholism, smoking, or excessive use of caffeine) except for regular exposure to sound from personal stereo devices. The fact that group 2 was older than group 1 underlines the importance of using personal stereo devices as a predisposing factor for tinnitus; previous reports have shown that the incidence of tinnitus increases with age.^1^ The discrepancy between a higher incidence of hypoacusis and preserved tone thresholds is probably related with temporary threshold shifts associated with exposure to loud sounds, which patients tended to mention in the clinical history.

The finding of a significantly higher mean threshold at 8 kHz in group 1 tinnitus patients (*p*=0.025) agrees with the theories that associate tinnitus with hearing loss.^15^, ^22^ However, as this frequency is not commonly affected in the initial phases of NIHL, unknown factors - other than noise exposure - may possibly be involved in the genesis of tinnitus in these patients; this possibility is also suggested by the previously described findings about TOAE. About 10% of patients with tinnitus have normal audiometries.^11^ In some of them, tinnitus may arise as a consequence of a central neuroplastic phenomenon following temporary hearing loss, such as occurs in otitis media or in sudden hearing loss. On the other hand, it is also possible that some of these patients have subclinical cochlear disease with normal tone thresholds; thus OAE may be an important tool for the early diagnosis of cochlear changes related to tinnitus, as has been mentioned in other papers. Prevention strategies should be implemented in these groups to prevent NIHL; OAE are an important tool for an early diagnosis.

## CONCLUSION

Tinnitus is more frequent in teenagers and young adults that regularly listen to music in personal stereo devices, and was associated with elevated audiometric thresholds at 8 kHz. Users of personal stereo devices had lower TOAE levels at 2 kHz.
